# Supplementary material for: Automated detection of lameness in sheep using machine learning approaches: novel insights into behavioural differences among lame and non-lame sheep
Source: R Soc Open Sci. 2020 Jan 15;7(1):190824. doi: 10.1098/rsos.190824 (PMC7029909; doi:10.1098/rsos.190824)
Supplement: Description of classification algorithms and complete feature set used in the study [file rsos190824supp1.docx]

**SUPPLEMENTARY MATERIAL**

**Methods**

**Description of the different classifiers used for lameness**

Random Forests^1,2^ are a type of ensemble learning method that combines multiple decision tree. The individual predictions of the tree models within the Random Forest are then combined into an overall classification decision, e.g. through the application of weights or a majority vote ^3^.

AdaBoost, short for Adaptive Boosting, is an ensemble learning methodology that combines the output of several individual models by majority vote to determine the final output of the ensemble classifier. Boosting is a step-wise procedure and a model is trained at each step. At every step, the weights assigned to the training samples are modified for the next step in a way so that previously misclassified training samples have their weights increased, whereas correctly classified samples have their weights are decreased ^4,5^.

A neural network is a set of interconnected layers, in which the inputs lead to outputs by a series of weighted edges and nodes. The weights on the edges are set when training the neural network on the input data. To compute the output of the network for any given input, a value is calculated for each node in the hidden layers and in the output layer. For each node, the value is set by calculating the weighted sum of the values of the nodes in the previous layer and applying an activation function to that weighted sum ^6^.

Support Vector Machine is a type of non-probabilistic classifier that that maps its inputs (i.e. the features characteristics) into a high-dimensional feature space, where each dimension represents one of the classification features. Support Vector Machines try to create a linear partition of the high-dimensional feature space into two subspaces. New data samples are later evaluated against this partition to determine their class membership ^7^.

The k-nearest neighbour classification algorithm (kNN) is a non-parametric method that uses the *k* closest samples in the training set to determine the class of an object. The classification decision is made by majority vote among its *k* nearest neighbours and to compute the k samples different distance measures can be used (e.g. Euclidean distance). Often, distances are weighted, so that the nearer neighbours contribute more than the ones further away (e. g. weight each with 1/d, where d is the distance of the object to the neighbour) ^8^.

**Feature selection**

| **Rank** | **Walking** | **Standing** | **Lying** |
| --- | --- | --- | --- |
| 1 | Spectral Entropy | Signal Area | Dominant Frequency |
| 2 | Zero Crossings | Interquartile Range | Spectral Entropy |
| 3 | Dominant Frequency | Spectral Area | Dominant Frequency |
| 4 | Dominant Frequency | Standard Deviation | Maximum |
| 5 | Spectral Entropy | Zero Crossings | Standard Deviation |
| 6 | 2nd Harmonic Frequency | Dominant Frequency | Minimum |
| 7 | Interquartile Range | 2nd Harmonic Frequency | Spectral Entropy |
| 8 | Signal Area | Spectral Entropy | Signal Area |
| 9 | Standard Deviation | 3rd Harmonic Frequency | Zero Crossings |
| 10 | Minimum | Interquartile Range | 2nd Harmonic Frequency |
| 11 | 2nd Harmonic Frequency | Minimum | 3rd Harmonic Frequency |
| 12 | Interquartile Range | Maximum | 2nd Harmonic Frequency |
| 13 | 3rd Harmonic Frequency | Signal Area | Interquartile Range |
| 14 | Maximum | Dominant Frequency | 3rd Harmonic Frequency |
| 15 | Maximum | Standard Deviation | Kurtosis |
| 16 | Spectral Area | 2nd Harmonic Frequency | Interquartile Range |
| 17 | Signal Area | Maximum | Signal Area |
| 18 | Standard Deviation | Spectral Entropy | Kurtosis |
| 19 | Minimum | Harmonic Ratio (First 20) | Spectral Area |
| 20 | Spectral Area | Harmonic Ratio | Standard Deviation |
| 21 | Zero Crossings | Minimum | Zero Crossings |
| 22 | Skewness | Zero Crossings | Skewness |
| 23 | 3rd Harmonic Frequency | 3rd Harmonic Frequency | Harmonic Ratio |
| 24 | Kurtosis | Kurtosis | Skewness |
| 25 | Skewness | Kurtosis | Harmonic Ratio (First 20) |
| 26 | Harmonic Ratio | Spectral Area | Maximum |
| 27 | Kurtosis | Harmonic Ratio | Minimum |
| 28 | Harmonic Ratio | Skewness | Harmonic Ratio |
| 29 | Harmonic Ratio (First 20) | Harmonic Ratio (First 20) | Harmonic Ratio (First 20) |
| 30 | Harmonic Ratio (First 20) | Mean | Mean |
| 31 | Mean | Skewness | Spectral Area |
| 32 | Mean | Mean | Mean |

**Table 1.** Full list of features ranked using ReliefF algorithm for walking, standing and lying. Light blue and dark blue colours represent acceleration magnitude difference based features with frequency domain and time domain, respectively. Light and dark green colours represents gyroscope magnitude difference based features with frequency domain and time domain, respectively.

**References**

[1]Breiman L. Random forests. *Mach. Learn*. **45,** 5–32 (2001).

[2] Cutler DR, *et al*. Random forest for classification in ecology. Ecology. **88,** 2783–2792 (2007).

[3] Hastie, T., Tibshirani, R. & Friedman, J. The elements of statistical learning. *Springer* (2009).

[4] Kégl, B. The return of AdaBoost. MH: multi-class Hamming trees. Preprint at <https://arxiv.org/abs/1312.6086> (2013).

[5] Freund, Y. & Schapire, R.E. A decision-theoretic generalization of on-line learning and an application to boosting. *J. Comput. Syst. Sci*. **55,** 119-139 (1997).

[6] Yadav, N., Yadav, A. & Kumar, M. An introduction to neural network methods for differential equations. *Springer* (2015)

[7] James, G., Witten, D., Hastie, T. & Tibshirani, R. An introduction to statistical learning: with application in R. *Springer* (2013).
