# Supplementary material for: Automated detection of lameness in sheep using machine learning approaches: novel insights into behavioural differences among lame and non-lame sheep
Source: R Soc Open Sci. 2020 Jan 15;7(1):190824. doi: 10.1098/rsos.190824 (PMC7029909; doi:10.1098/rsos.190824)
Supplement: Python Code for Lameness classification machine learning algorithms [file rsos190824supp2.docx]

**SUPPLEMENTARY MATERIAL**

**Classification code written in Python**

Script for the classification algorithm written in Python

import pandas as pd

from skrebate import ReliefF

from sklearn.ensemble import RandomForestClassifier, AdaBoostClassifier

from sklearn.neighbors import KNeighborsClassifier

from sklearn.svm import SVC

from sklearn.neural_network import MLPClassifier

from sklearn.model_selection import cross_validate, StratifiedKFold

from sklearn.metrics import accuracy_score, precision_score, recall_score

from sklearn.metrics import confusion_matrix, make_scorer, f1_score

from sklearn.model_selection import cross_val_predict

#%% scoring functions

def tn(y_true, y_pred):

return confusion_matrix(y_true, y_pred)[0, 0]

def fp(y_true, y_pred):

return confusion_matrix(y_true, y_pred)[0, 1]

def fn(y_true, y_pred):

return confusion_matrix(y_true, y_pred)[1, 0]

def tp(y_true, y_pred):

return confusion_matrix(y_true, y_pred)[1, 1]

#%% read in & pre-process featureset

# specify file path & read file

featureset2018_p = 'INSERT PATH HERE'

fs2018 = pd.read_csv(featureset2018_p)

# generate binary Lameness column

fs2018.loc[fs2018.LMS < 2, 'Lameness'] = 0

fs2018.loc[fs2018.LMS > 1, 'Lameness'] = 1

#%% Define Label & Select relevant columns: AccMag_g_dff & GyrMag_diff

# initialise column list

diff_feature_columns = ['SheepID', 'RecordID', 'Day', 'WSL', 'Lameness']

# only use columns that are based on AccMag_G and GyrMag Diff columns

for col in fs2018.columns:

if (('AccMag_g' in col) or ('GyrMag' in col)) & ('diff' in col):

diff_feature_columns.append(col)

# filter featureset

RelevantSubset = fs2018[diff_feature_columns]

#%% variables for classificaiton

# diverse range of models

models = [RandomForestClassifier(n_estimators=250, class_weight="balanced"),

SVC(), MLPClassifier(), KNeighborsClassifier(5),

AdaBoostClassifier(n_estimators=100)]

names = ['RF', 'SVM', 'MLP', 'KNC', 'AdaBoost']

# measures to extract

measures = ['accuracy', 'precision', 'recall', 'f1', 'specificity']

# stratified 10-fold cross-validation

skf = StratifiedKFold(10, shuffle=True)

# specify scoring functions to use

scoring = {'accuracy': make_scorer(accuracy_score),

'precision': make_scorer(precision_score),

'recall': make_scorer(recall_score),

'f1': make_scorer(f1_score),

'tp' : make_scorer(tp), 'tn' : make_scorer(tn),

'fp' : make_scorer(fp), 'fn' : make_scorer(fn)}

# %% initialise result DF

# result df column names

colnames = ['WSL', 'Features']

for col in [f'{i}_{measure}' for i in names for measure in measures]:

colnames.append(col)

#%% Classifier Pipeline - ReleiefF

# initialise result df

Train = pd.DataFrame(columns=colnames)

Test = pd.DataFrame(columns=colnames)

# iterate over activities

for wsl in [1,2,3]:

print(wsl)

# filter featureset for relevant behaviour

RSwsl = RelevantSubset[RelevantSubset.WSL == wsl].copy()

Xwsl = RSwsl.drop(['SheepID', 'RecordID', 'Day', 'WSL', 'Lameness'],

axis=1).copy()

y = RelevantSubset[RelevantSubset.WSL == wsl].Lameness.copy()

# Feature Ranking

print('apply ReliefF')

RelF = ReliefF()

RelF.fit(Xwsl.values, y.values)

Feature_Ranking = Xwsl.columns[RelF.top_features_]

print(Feature_Ranking)

for nf in range(1,33):

print(f'{nf} feature(s)')

print(Feature_Ranking[:nf])

X = Xwsl[Feature_Ranking[:nf]]

# model building

training = [wsl, nf]

testing = [wsl, nf]

for est, name in zip(models, names):

print(name)

cv_res = cross_validate(est, X, y, cv=skf,

scoring=scoring, return_train_score=True)

RSwsl[f'{name}_predictions_{nf}'] = cross_val_predict(est, X, y, cv=skf)

# store cv results in result vector

print('append results')

for measure in measures:

if measure == 'specificity':

continue

training.append(cv_res[f'train_{measure}'].mean())

testing.append(cv_res[f'test_{measure}'].mean())

training.append((cv_res['train_tn'] / (cv_res['train_tn'] + cv_res['train_fp'])).mean())

testing.append((cv_res['test_tn'] / (cv_res['test_tn'] + cv_res['test_fp'])).mean())

Train.loc[len(Train)] = training

Test.loc[len(Test)] = testing

print()

RSwsl.to_csv('INSERT PATH HERE', index=False)

print()

Train.to_csv('INSERT PATH HERE', index=False)

Test.to_csv('INSERT PATH HERE', index=False)
