# Supplementary material for: Automated detection of lameness in sheep using machine learning approaches: novel insights into behavioural differences among lame and non-lame sheep
Source: R Soc Open Sci. 2020 Jan 15;7(1):190824. doi: 10.1098/rsos.190824 (PMC7029909; doi:10.1098/rsos.190824)
Supplement: Lameness classification approach including test and train data across the folds and percentage misclassification [file rsos190824supp3.docx]

**SUPPLEMENTARY MATERIAL**

**Lameness classification approach**

An individual classifier model was developed within each of the different activities (walking, standing and lying). Classification performance was evaluated using 10-fold cross-validation, a commonly used methodology that provides robust evaluation in classification model performance. Stratification based on the lameness class was applied during the splitting to ensure that class representations in each of the subsets was equal to the original dataset. Tables 2-4 shown the average and standard deviation percentage of data across all the folds on the testing set for each individual sheep. This tables were obtained using the best classifier model which was a Random Forest model with 10 feature characteristics. Using the same model misclassification percentages for each individual sheep are shown in Tables 5-7.

| **WSL** | **UID** | **Mean (in %)** | **SD (in %)** | **Lameness** | **Data**  **Points** | **% of Total**  **(per behaviour)** |
| --- | --- | --- | --- | --- | --- | --- |
| **Walking** | 2016_1 | 10 | 0.14 | Lame | 682 | 11 |
|  | 2016_2 | 10 | 0.29 | Lame | 328 | 5 |
|  | 2016_3 | 10 | 0.26 | Lame | 356 | 6 |
|  | 2016_6 | 10 | 0.17 | Non-Lame | 324 | 5 |
|  | 2016_7 | 10 | 0.14 | Non-Lame | 362 | 6 |
|  | 2016_8 | 10 | 0.18 | Non-Lame | 335 | 5 |
|  | 2016_10 | 11 | 0.53 | Lame | 322 | 5 |
|  | 2016_11 | 9 | 0.18 | Lame | 325 | 5 |
|  | 2016_12 | 10 | 0.16 | Lame | 324 | 5 |
|  | 2016_13 | 10 | 0.09 | Non-Lame | 674 | 11 |
|  | 2016_15 | 9 | 0.14 | Lame | 335 | 5 |
|  | 2016_18 | 10 | 0.23 | Non-Lame | 324 | 5 |
|  | 2017_4 | 10 | 0.22 | Lame | 256 | 4 |
|  | 2017_6 | 10 | 0.54 | Non-Lame | 266 | 4 |
|  | 2017_7 | 9 | 0.37 | Non-Lame | 177 | 3 |
|  | 2017_9 | 10 | 0.42 | Non-Lame | 256 | 4 |
|  | 2017_16 | 10 | 0.07 | Lame | 442 | 7 |
|  | 2017_18 | 11 | 0.11 | Non-Lame | 256 | 4 |

**Table 2**.

| **WSL** | **UID** | **Mean (in %)** | **SD (in %)** | **Lameness** | **Data**  **Points** | **% of Total**  **(per behaviour)** |
| --- | --- | --- | --- | --- | --- | --- |
| **Standing** | 2016_1 | 10 | 0.05 | Lame | 1371 | 18 |
|  | 2016_2 | 10 | 0.17 | Lame | 549 | 7 |
|  | 2016_3 | 9 | 0.08 | Lame | 588 | 8 |
|  | 2016_6 | 10 | 0.10 | Non-Lame | 923 | 12 |
|  | 2016_7 | 10 | 0.44 | Non-Lame | 159 | 2 |
|  | 2016_8 | 11 | 1.19 | Non-Lame | 45 | 1 |
|  | 2016_10 | 9 | 0.99 | Lame | 108 | 1 |
|  | 2016_11 | 11 | 0.77 | Lame | 102 | 1 |
|  | 2016_12 | 11 | 0.84 | Lame | 94 | 1 |
|  | 2016_13 | 10 | 0.08 | Non-Lame | 558 | 8 |
|  | 2016_15 | 11 | 0.34 | Lame | 95 | 1 |
|  | 2016_18 | 10 | 0.05 | Non-Lame | 595 | 8 |
|  | 2017_6 | 10 | 0.10 | Non-Lame | 811 | 11 |
|  | 2017_7 | 10 | 0.11 | Non-Lame | 731 | 10 |
|  | 2017_16 | 10 | 0.10 | Lame | 684 | 9 |

**Table 3.**

| **WSL** | **UID** | **Mean (in %)** | **SD (in %)** | **Lameness** | **Data**  **Points** | **% of Total**  **(per behaviour)** |
| --- | --- | --- | --- | --- | --- | --- |
| **Lying** | 2016_1 | 10 | 0.04 | Lame | 1153 | 18 |
|  | 2016_2 | 10 | 0.22 | Lame | 629 | 10 |
|  | 2016_3 | 10 | 0.01 | Lame | 1224 | 19 |
|  | 2016_6 | 10 | 0.10 | Non-Lame | 513 | 8 |
|  | 2016_7 | 10 | 0.05 | Non-Lame | 507 | 8 |
|  | 2016_8 | 10 | 0.10 | Non-Lame | 479 | 8 |
|  | 2016_10 | 10 | 0.08 | Lame | 759 | 12 |
|  | 2016_12 | 10 | 0.40 | Lame | 190 | 3 |
|  | 2016_13 | 10 | 0.07 | Non-Lame | 755 | 12 |
|  | 2016_15 | 10 | 0.18 | Lame | 112 | 2 |
|  | 2017_7 | 12 | 1.38 | Non-Lame | 17 | 0 |
|  | 2017_16 | 13 | 4.91 | Lame | 9 | 0 |

**Table 4**

**Tables 2-4.** Percentage of data per used in the testing dataset for each sheep and within each behaviour. UID represents the year (2016 or 2017) and the sheep ID. The mean was computed as the average percentage of the data used in the testing dataset over all the different folds for each individual sheep. Similarly, the standard deviation (SD) was computed using the standard deviation of the data used in the testing dataset over all the different folds for each individual sheep. Data points indicate the number of total data points collected for each individual sheep within each behaviour. The percentage of total per behaviour column represents the number of data samples for each individual sheep over the total number of samples within each activity.

| **WSL** | **UID** | **Misclassified 7s samples (%)** | **Lameness** | **Data**  **Points** | **% of Total**  **(per behaviour)** |
| --- | --- | --- | --- | --- | --- |
| **Walking** | 2016_1 | 13.93 | Lame | 682 | 11 |
|  | 2016_2 | 3.05 | Lame | 328 | 5 |
|  | 2016_3 | 4.21 | Lame | 356 | 6 |
|  | 2016_6 | 12.04 | Non-Lame | 324 | 5 |
|  | 2016_7 | 26.80 | Non-Lame | 362 | 6 |
|  | **2016_8** | **55.52** | **Non-Lame** | **335** | **5** |
|  | **2016_10** | **50.93** | **Lame** | **322** | **5** |
|  | 2016_11 | 18.77 | Lame | 325 | 5 |
|  | 2016_12 | 10.19 | Lame | 324 | 5 |
|  | 2016_13 | 24.63 | Non-Lame | 674 | 11 |
|  | 2016_15 | 44.18 | Lame | 335 | 5 |
|  | 2016_18 | 20.06 | Non-Lame | 324 | 5 |
|  | 2017_4 | 19.14 | Lame | 256 | 4 |
|  | 2017_6 | 33.83 | Non-Lame | 266 | 4 |
|  | 2017_7 | 44.63 | Non-Lame | 177 | 3 |
|  | 2017_9 | 40.63 | Non-Lame | 256 | 4 |
|  | 2017_16 | 12.22 | Lame | 442 | 7 |
|  | 2017_18 | 29.30 | Non-Lame | 256 | 4 |

**Table 5**.

| **WSL** | **UID** | **Misclassified 7s samples (%)** | **Lameness** | **Data**  **Points** | **% of Total**  **(per behaviour)** |
| --- | --- | --- | --- | --- | --- |
| **Standing** | 2016_1 | 7.22 | Lame | 1371 | 18 |
|  | 2016_2 | 14.21 | Lame | 549 | 7 |
|  | 2016_3 | 11.73 | Lame | 588 | 8 |
|  | 2016_6 | 16.58 | Non-Lame | 923 | 12 |
|  | **2016_7** | **79.25** | **Non-Lame** | **159** | **2** |
|  | **2016_8** | **55.56** | **Non-Lame** | **45** | **1** |
|  | 2016_10 | 11.11 | Lame | 108 | 1 |
|  | **2016_11** | **57.84** | **Lame** | **102** | **1** |
|  | 2016_12 | 11.70 | Lame | 94 | 1 |
|  | **2016_13** | **62.54** | **Non-Lame** | **558** | **8** |
|  | 2016_15 | 15.79 | Lame | 95 | 1 |
|  | 2016_18 | 30.76 | Non-Lame | 595 | 8 |
|  | 2017_6 | 2.47 | Non-Lame | 811 | 11 |
|  | 2017_7 | 7.11 | Non-Lame | 731 | 10 |
|  | 2017_16 | 19.15 | Lame | 684 | 9 |

**Table 6**.

| **WSL** | **UID** | **Misclassified 7s samples (%)** | **Lameness** | **Data**  **Points** | **% of Total**  **(per behaviour)** |
| --- | --- | --- | --- | --- | --- |
| **Lying** | 2016_1 | 1.91 | Lame | 1153 | 18 |
|  | 2016_2 | 1.91 | Lame | 629 | 10 |
|  | 2016_3 | 8.09 | Lame | 1224 | 19 |
|  | **2016_6** | **58.09** | **Non-Lame** | **513** | **8** |
|  | 2016_7 | 21.70 | Non-Lame | 507 | 8 |
|  | 2016_8 | 29.85 | Non-Lame | 479 | 8 |
|  | 2016_10 | 0.66 | Lame | 759 | 12 |
|  | 2016_12 | 1.58 | Lame | 190 | 3 |
|  | 2016_13 | 40.79 | Non-Lame | 755 | 12 |
|  | 2016_15 | 0.00 | Lame | 112 | 2 |
|  | **2017_7** | **94.12** | **Non-Lame** | **17** | **0** |
|  | 2017_16 | 0.00 | Lame | 9 | 0 |

**Table 7**.

**Tables 5-7.** Percentage of the misclassification for each sheep at 7s window level and within each behaviour. UID represents the year (2016 or 2017) and the sheep ID. In bold are the sheep with a misclassification based on threshold computed which indicates that the individual sheep was incorrectly classified.
